# Economic costs at age five associated with very preterm birth: multinational European cohort study

**DOI:** 10.1038/s41390-021-01769-z

**Published:** 2021-11-12

**Authors:** Sung Wook Kim, Lazaros Andronis, Anna-Veera Seppänen, Adrien M. Aubert, Jennifer Zeitlin, Henrique Barros, Elizabeth S. Draper, Stavros Petrou, on behalf of the SHIPS Research Group, J. Lebeer, P. Van Reempts, E. Bruneel, E. Cloet, A. Oostra, E. Ortibus, I. Sarrechia, K. Boerch, P. Pedersen, L. Toome, H. Varendi, M. Männamaa, P. Y. Ancel, A. Burguet, P. H. Jarreau, V. Pierrat, P. Truffert, R. F. Maier, M. Zemlin, B. Misselwitz, L. Wohlers, M. Cuttini, I. Croci, V. Carnielli, G. Ancora, G. Faldella, F. Ferrari, A. van Heijst, C. Koopman-Esseboom, J. Gadzinowski, J. Mazela, A. Montgomery, T. Pikuła, H. Barros, R. Costa, C. Rodrigues, U. Aden, E. S. Draper, A. Fenton, S. J. Johnson, S. Mader, N. Thiele, J. M. Pfeil, S. Petrou, S. W. Kim, L. Andronis, J. Zeitlin, C. Bonnet, R. El Rafei, A. V. Seppänen, A. M. Aubert

**Affiliations:** 1 Nuffield Department of Primary Care Health Sciences, University of Oxford, Oxford, UK; 2 Warwick Clinical Trials Unit, Warwick Medical School, University of Warwick, Coventry, UK; 3 CRESS, Obstetrical Perinatal and Pediatric Epidemiology Research Team, EPOPé, INSERM, INRAE, Université de Paris, F-75004 Paris, France; 4 EPIUnit-Instituto de Saúde Pública da Universidade do Porto, Porto, Portugal; 5 Department of Health Sciences, University of Leicester, Leicester, UK; 6 Department of Family Medicine & Population Health (FAMPOP), Disability Studies, Faculty of Medicine & Health Sciences, University of Antwerp, Antwerp, Belgium; 7 Laboratory of Experimental Medicine and Pediatrics, Division of Neonatology, University of Antwerp, Antwerp, Belgium; 8 Study Centre for Perinatal Epidemiology Flanders, Brussels, Belgium; 9 Centre for Developmental Disabilities, Neonatal Intensive Care, Oost Limburg Hospital, Genk, Belgium; 10 Faculteit Geneeskunde en Farmacie, Vrije Universiteit Brussel, Brussels, Belgium; 11 Paediatric Neurology, Universitair Ziekenhuis Brussels, Brussels, Belgium; 12 Centre for Developmental Disabilities, Ghent University Hospital, Ghent, Belgium; 13 Centre for Developmental Disabilities, Leuven University Hospital, Leuven, Belgium; 14 Department of Neuropediatrics, University of Leuven, Leuven, Belgium; 15 Centre for Developmental Disabilities, Antwerp University Hospital, Antwerp, Belgium; 16 Department of Primary & Interdisciplinary Care, Disability Studies, Faculty of Medicine, University of Antwerp, Antwerp, Belgium; 17 Department of Paediatrics, Hvidovre Hospital, Copenhagen University Hospital, Hvidovre, Denmark; 18 Department of Neonatology, Hvidovre Hospital, Hvidovre, Denmark; 19 Tallinn Children’s Hospital, Tallinn, Estonia; 20 University of Tartu, Tartu, Estonia; 21 Tartu University Hospital, University of Tartu, Tartu, Estonia; 22 Department of Paediatrics, Institute of Clinical Medicine, University of Tartu, Tartu, Estonia; 23 Division of Pediatrics 2, Hôpital du Bocage, INSERM CIE1, CHRU Dijon, Université de Dijon, Dijon, France; 24 Université Paris Descartes and Assistance Publique Hôpitaux de Paris, Hôpitaux Universitaire Paris Centre Site Cochin, DHU Risks in Pregnancy, Service de Médecine et Réanimation néonatales de Port-Royal, Paris, France; 25 CHU Lille, Department of Neonatal Medicine, Jeanne de Flandre Hospital, 59000 Lille, France; 26 Children’s Hospital, University Hospital, Philipps University Marburg, Marburg, Germany; 27 University Medical Center, Homburg, Germany; 28 Institute of Quality Assurance Hesse, Eschborn, Germany; 29 Clinical Care and Management Innovation Research Area, Bambino Gesù Children’s Hospital, IRCCS, Rome, Italy; 30 Maternal and Child Health Institute, Marche University and Salesi Hospital, Ancona, Italy; 31 Neonatal Intensive Care Unit, Ospedale degli Infermi, Rimini, Italy; 32 Neonatal Intensive Care Unit, University Hospital S. Orsola-Malpighi, Bologna, Italy; 33 Department of Pediatrics and Neonatology, Modena University Hospital, Modena, Italy; 34 Department of Neonatology, Radboud University Medical Center, Nijmegen, the Netherlands; 35 Department of Neonatology, Wilhelmina Children’s Hospital, Utrecht, the Netherlands; 36 Department of Neonatology, Poznan University of Medical Sciences, Poznan, Poland; 37 Department of Neonatology and Neonatal Infectious Diseases, Poznan University of Medical Sciences, Poznan, Poland; 38 Katedra i Klinika Neonatologii, Uniwersytet Medyczny im. Karola Marcinkowskiego w Poznaniu, Poznan, Poland; 39 Department of Women’s and Children’s Health, Karolinska Institutet, Stockholm, Sweden; 40 Newcastle University, Newcastle upon Tyne, UK; 41 European Foundation for the Care of Newborn Infants (EFCNI), Munich, Germany; 42 Warwick Medical School, University of Warwick, Coventry, UK

## Abstract

**Background:**

This study aims to estimate the economic costs of care provided to children born very preterm and extremely preterm across 11 European countries, and to understand what perinatal and socioeconomic factors contribute to higher costs.

**Methods:**

Generalised linear modelling was used to explore the association between perinatal and sociodemographic characteristics and total economic costs (€, 2016 prices) during the fifth year of life.

**Results:**

Lower gestational age was associated with increased mean societal costs of €2755 (*p* < 0.001), €752 (*p* < 0.01) and €657 (*p* < 0.01) for children born at < 26, 26–27 and 28–29 weeks, respectively, in comparison to the reference group born at 30–31 weeks. A sensitivity analyses that excluded variables (BPD, any neonatal morbidity and presence of congenital anomaly) plausibly lying on the causal pathway between gestational age at birth and economic outcomes elevated incremental societal costs by €1482, €763 and €144 at < 26, 26–27 and 28–29 weeks, respectively, in comparison to the baseline model.

**Conclusion:**

This study provides new evidence about the main cost drivers associated with preterm birth in European countries. Evidence identified by this study can act as inputs within cost-effectiveness models for preventive or treatment interventions for preterm birth.

**Impact:**

- What is the key message of your article? This study provides new evidence about the magnitude and drivers of economic costs associated with preterm birth in European countries.
- What does it add to the existing literature? Lower gestational age is associated with increased mean societal costs during mid-childhood with indirect costs representing a key driver of increased costs.
- What is the impact? For policy makers, this study adds to sparse evidence about the main cost drivers associated with preterm birth in European countries beyond the first 2 years of life.

## Introduction

Preterm birth, defined by the World Health Organisation as childbirth before 37 completed weeks’ gestation,^1^ remains a major cause of childhood morbidity and mortality around the world.^2^ Children born very preterm (VPT) are more likely to suffer from cerebral palsy, sensory deficits, learning disabilities and respiratory illnesses compared to children born at term, and face an increased risk of death.^3^ The presence and severity of health and developmental issues associated with preterm birth is inversely related to gestational age at birth, with VPT (i.e., 28–31 weeks) and extremely preterm (EPT) (i.e., before 28 gestational weeks) born children being at greater risk of adverse outcomes.^4^

Intensified efforts to put in place cost-effective networks of services^5,6^ for VPT and EPT born children has created a need to understand what health care and broader services those born preterm need and use, and how much these services cost. In Europe, available data draw a heterogeneous picture of preterm birth trends: while in some countries preterm birth levels have remained stable or declined, a number of countries have experienced an increase in the number of infants born preterm.^7–9^ As morbidity rates and developmental problems are high for this group,^10^ there is a pressing need to quantify the economic costs associated with very and EPT birth, and to understand the factors that contribute to elevated costs.

Previous European-wide studies of VPT and EPT birth have largely focussed on its epidemiological features. An earlier multinational study, Models of OrganiSing Access to Intensive Care for VPT births (MOSAIC),^11^ followed up a large cohort of 7222 preterm infants born between 22 and 31 weeks’ gestational age in 2003 across 10 European countries and reported wide variations in mortality. A study by Field et al.^12^ concluded that variation in the rate of VPT delivery had a major impact on death rates in 10 European regions. Zeitlin et al.^13^ studied variations in preterm birth rates across 11 European countries and reported wide variations over the past 15 years.

Despite a growing understanding of mortality and morbidity following VPT birth across Europe,^11,13^ evidence on its economic consequences is lacking. There are no consistent data with respect to costs or cost burden of VPT birth across European countries.^7^ Moreover, a relatively small number of studies have estimated the economic costs associated with preterm birth in individual countries. Khan et al.^14^ reported the economic costs associated with preterm birth in the UK, but focussed solely on moderate and late preterm births. This study recruited approximately 2400 infants in the East Midlands and estimated incremental costs between birth and 24 months’ corrected age of £12,037 (€ 13,875) and £5823 (€ 6712) for children born moderate and late preterm, respectively (2010–2011 prices). Petrou et al.^15^ reported the cumulative cost (£17,819) of hospital inpatient admissions during the first 10 years of life for children born EPT (1998/1999 prices). In the US, the mean medical care cost up to 5 years of age associated with preterm birth was estimated at $31,290 (2005 prices).^16^

Apart from the fact that existing evidence is limited to individual countries, the available literature is largely limited to economic costs incurred by the health services.^17–19^ Rarely have consequences accruing to other sectors of the economy or to individuals been captured or quantified.^14,20^ A recent systematic review of the economic consequences of preterm birth revealed that only eight studies had quantified economic costs borne outside of the health sector post initial discharge.^21^ Moreover, several categories of economic costs, such as additional costs borne by families as a result of modifications to their everyday activities, have been traditionally overlooked.

The aims of this study were therefore two-fold: first, to provide estimates of the economic costs incurred by the health care system, caregivers and society as a whole attributable to VPT and EPT births across 11 European countries, and second to identify which of a series of clinical and socioeconomic determinants is associated with elevated economic costs.

## Methods

### Study population

Individual-level data related to children born VPT or EPT, as well as the sociodemographic characteristics of their parents, were collected as part of the Effective Perinatal Intensive care in Europe (EPICE) and Screening to improve Health In very Preterm infantS (SHIPS) studies. A detailed description of the design, outcome measures and data collection processes for the EPICE and SHIPS studies can be found in Zeitlin et al.^22^ Briefly, the EPICE and SHIPS studies constituted and followed up an area-based cohort of children born between 22^+0^ and 31^+6^ weeks of gestation in 2011/2012 in all maternity units in 19 regions across 11 European countries: Belgium (Flanders); Denmark (Eastern Region); Estonia (entire country); France (Burgundy, Ile-de-France and the Northern region); Germany (Hesse and Saarland); Italy (Emilia-Romagna, Lazio and Marche); the Netherlands (Central and Eastern region); Poland (Wielkopolska); Portugal (Lisbon and Northern region); Sweden (greater Stockholm) and the United Kingdom (East Midlands, Northern, and Yorkshire and the Humber regions). Parental questionnaires for all children and neurodevelopmental assessments for children born at < 28 weeks were used as the primary vehicles for data collection.

### Costing methodology

A retrospective assessment of economic costs incurred during the fifth year of participating children’s lives was embedded within the SHIPS cohort study design. The estimation of costs followed a standard approach, where use of a service (resource use) is weighted by a value reflecting the opportunity cost, or ‘price’, of the service (unit cost). The two stages in estimating economic costs, namely measurement and valuation of resource use, are described below.

### Measurement of resource use

When participating children reached their fifth year of age, parents were invited to complete a self-reported questionnaire designed to collect information on their children’s health, development and use of health care and other services during the previous year, the parents’ circumstances, health, wellbeing and restrictions imposed by their preterm child’s health. The survey questionnaire attempted to capture how much of the additional restrictions were directly attributable to their child’s health. The questionnaire was piloted to ascertain its acceptability, comprehension and reliability, and reminder letters were sent to parents to increase the response and completion rates.

Data from the SHIPS questionnaire at 5 years of age provided detailed information about children’s service use and expenses over the preceding year, including: (i) use of hospital outpatient services such as contacts with paediatricians, accident and emergency departments, ear, nose and throat specialists, eye specialists, speech/language therapists, psychologists, psychiatrists, physiotherapists or motor developmental therapists, respiratory or asthma specialists and routine follow-up services for preterm born children; (ii) hospital inpatient stays; (iii) use of community health and social services such as contacts with general practitioners, dieticians, health visitors, school nurses, occupational therapists and early childhood intervention services; and (iv) additional expenses incurred by parents resulting from caring for their child, including equipment (e.g., wheelchairs) purchased and, separately, duration of work absences as a result of the child’s health. The costing analysis was performed from two perspectives: a health and social care provider perspective and, separately, a societal perspective.^23^ The health and social care provider perspective encompassed the costs associated with use of hospital outpatient services, hospital inpatient stays and use of community health and social services (categories (i), (ii) and (iii) above), whilst the societal perspective encompassed all categories of service use as well as relevant expenses incurred by parents and valuations of work absences (based on categories (i), (ii), (iii) and (iv) above).^24^

### Valuation of resource use

Lack of central resources or publications listing unit costs for different episodes or aspects of care in many of the participating countries made it necessary to employ an iterative approach to valuing resource inputs. The sources are described in Appendix 1. Unit costs were taken from national sources or publications where available. When such sources were not available, information was extracted from the relevant peer-reviewed literature, typically costing exercises within published economic evaluations or cost analyses or through the data collection systems of SHIPS collaborating centres. For example, for the UK, unit costs were mainly sourced from the Personal Social Services Research Unit’s (PSSRU) Unit Costs of Health and Social Care compendium^25^ and additional publications were searched for unit costs not listed in the PSSRU reports. The collected cost items were validated with local health economists and project investigators in each region.

Unit costs were converted to the reference year of 2016, the latest year for which indices were available at the beginning of this study, using a cost conversion tool.^26^ Country-specific currencies were converted to EUR (€) using purchasing power parity (PPP) values published by the Organisation for Economic Co-operation and Development (OECD). Work absences as a result of the health of the child were valued using nationally specific earnings data published by the OECD.^27^

### Statistical analysis

Differences in characteristics between children born VPT and EPT were analysed using *χ*^2^ tests for categorical variables and *t*-tests for continuous variables. Within country, generalised linear model (GLM) regression was carried out to explore the association between various perinatal and sociodemographic characteristics and total economic costs during the fifth year of life. GLM regression is recommended for dealing with skewness in the distribution of outcome variables,^28,29^ which is typically the case with cost data. Various model specifications and their goodness-of-fit were explored by generating and comparing Akaike Information Criterion (AIC) estimators. The AIC is an estimator for prediction errors of statistical models, and models with lower AIC scores are preferred.^30^ This led to the selection of a negative binomial distribution and a log link for the GLM regressions.^30,31^ We performed the Kolmogorov–Smirnov test for equality of distribution functions (*p* < 0.001).

Covariates that were controlled for in the GLM regressions were selected based on published evidence and included: (i) clinical variables such as small for gestational age (SGA) status (birthweight < 3rd, 3rd–9th, ≥10th centile for gestational age), presence of a congenital anomaly (yes or no), diagnosis of bronchopulmonary dysplasia (BPD) [defined as oxygen or respiratory support at 36 weeks’ GA] (yes or no), sex of baby (female or male), any neonatal morbidity (yes or no) [defined as having at least one of the following symptoms: intraventricular haemorrhage grades III–IV, cystic periventricular leukomalacia, retinopathy of prematurity stages III–V or necrotising enterocolitis needing surgery], multiplicity status (singletons, twins, triplets and quadruplets), and parity (nulliparous, multiparous)^14^ and (ii) sociodemographic variables including maternal country of birth (European born or non-European born),^32^ household employment status (at least one parent unemployed),^23^ maternal age (< 25, 25–34, > 34 years old) at delivery and maternal education level (high school or less, university).^32^ We also conducted sensitivity analyses that excluded: (1) the BPD and any neonatal morbidity variables; (2) the BPD, any neonatal morbidity and presence of a congenital anomaly variables; and (3) the BPD, any neonatal morbidity, presence of a congenital anomaly and employment status variables; as these variables plausibly lie on the causal pathway between preterm birth status and total economic costs. Inverse probability weights were also embedded within the regression analyses to account for attrition during follow-up.

In addition, a multi-level mixed effects GLM was employed to pool the results of the country-specific regressions across the country level.^33^ GLM mixed effects models are extensions of GLMs that allow for the estimation of random effects at the cluster level. This model included both fixed effects and random effects: the fixed effects were regression coefficients at the individual (here, country) level while the random effects were presented as variances at the cluster level.^34^ All analyses were conducted using STATA 16 (Stata-Corp, College Station, TX).

## Results

Of 7900 live births in the EPICE/SHIPS cohort, 5378 children (approximately 68% of the cohort) were born VPT and 2522 children were born EPT. Gestational age, SGA below the 10th centile and in-hospital deaths after live births were statistically significantly different between the VPT and EPT born children (*p* < 0.001). Specifically, the proportion of SGA children below the 10th decile at birth was significantly higher in EPT children, and so was the proportion of live born children dying before hospital discharge. Singletons accounted for 68% (*N* = 3634) of all VPT children and 71% (*N* = 1803) of all EPT children, whereas twins accounted for 29% (*N* = 1549) and 25% (*N* = 635) of the VPT and EPT children, respectively.

The 5-year follow-up rate using the denominator of all live and eligible children (*N* = 6759) was 54.5% (*N* = 3687). Of the 3687 children followed-up at 5 years, singletons accounted for 67% (*N* = 1776) of VPT children and 72% (*N* = 733) of EPT children, whilst twins accounted for 30% (*N* = 806) and 25% (*N* = 256) of the VPT and EPT children, respectively. The mean gestational age at birth was 29.8 weeks (SE = 0.02) for VPT children and 25.9 (SE = 0.03) weeks for EPT children. Economic costs during the fifth year of life were estimated for the children who were followed up at 5 years and for whom questionnaires were completed (*N* = 3529). A flowchart of the selection process for participants is presented in Appendix 2.

Table 1 provides descriptive statistics for child characteristics in the SHIPS cohort by country and gestational age group, and concomitant 5-year follow-up rates. France had the highest number of preterm born children in the cohort (1307), whilst Estonia had the lowest number (153). Estonia had the highest follow-up rate at 5 years of age for VPT children (91.4%), whilst the UK had the lowest follow-up rate for VPT children (25.3%). Over half of the cohort was male in all countries except for Estonia, Italy and the Netherlands. Table 1 highlights variations in participant characteristics and 5-year follow-up rates across countries.

**Table 1:** Summary statistics of participant characteristics (the entire cohort of live births), by country.

	Belgium	Denmark	Estonia	France	Germany	Italy	Netherlands	Poland	Portugal	UK	Sweden
*Infants (N)*											
VPT^a^	549	210	105	883	492	792	255	199	479	1229	185
EPT^b^	203	141	48	424	266	342	138	117	245	516	82
*Gestational age, completed weeks, median (IQR)*											
VPT	30 (29–31)	30 (29–31)	30 (29–31)	30 (29–31)	30 (29–31)	30 (29–31)	30 (29–31)	30 (29–31)	30 (29–31)	30 (29–31)	30 (29–31)
EPT	25 (24–27)	26 (25–27)	26 (25–27)	26 (25–27)	25 (24–27)	26 (24–27)	26 (24–26)	25 (23–26)	26 (25–27)	26 (24–27)	25 (24–27)
*Gestational age, mean (SD)*											
VPT	29.9 (1.1)	29.7 (1.1)	29.9 (1.0)	29.8 (1.1)	29.8 (1.1)	29.8 (1.1)	30.0 (1.0)	30.0 (1.1)	29.8 (1.1)	29.8 (1.1)	29.6 (1.1)
EPT	25.3 (1.4)	25.6 (1.5)	25.9 (1.2)	25.5 (1.4)	25.2 (1.6)	25.4 (1.5)	25.4 (1.4)	24.8 (1.7)	25.8 (1.2)	25.3 (1.4)	25.0 (1.6)
*Male, N (%)*											
VPT	304 (55.4)	113 (53.8)	59 (56.2)	474 (53.7)	261 (53.0)	400 (50.5)	128 (50.2)	115 (57.8)	278 (58.0)	681 (55.4)	100 (54.1)
EPT	122 (60.1)	72 (51.1)	21 (43.8)	227 (53.5)	146 (54.9)	163 (47.7)	67 (48.6)	61 (52.1)	145 (59.2)	284 (55.0)	45 (54.9)
*Singleton, N (%)*											
VPT	328 (59.7)	135 (64.3)	75 (71.4)	597 (67.6)	304 (61.8)	515 (65.0)	187 (73.3)	153 (76.9)	321 (67.0)	897 (73.1)	122 (65.9)
EPT	136 (67.0)	73 (51.8)	44 (91.7)	284 (67.0)	183 (68.8)	253 (74.0)	87 (63.0)	98 (83.8)	179 (73.1)	403 (78.1)	63 (76.8)
*Twins, N (%)*											
VPT	210 (38.3)	66 (31.4)	21 (20.0)	262 (29.7)	155 (31.5)	237 (29.9)	62 (24.3)	43 (21.6)	156 (32.6)	283 (23.1)	54 (29.2)
EPT	61 (30.0)	59 (41.8)	4 (8.3)	130 (30.7)	67 (25.2)	71 (20.8)	39 (28.3)	19 (16.2)	59 (24.1)	110 (21.3)	16 (19.5)
*Triplets, N (%)*											
VPT	11 (2.0)	9 (4.3)	9 (8.6)	24 (2.7)	33 (6.7)	40 (5.1)	6 (2.4)	3 (1.5)	2 (0.4)	47 (3.8)	9 (4.9)
EPT	6 (3.0)	9 (6.4)	0 (0.00)	10 (2.4)	16 (6.0)	18 (5.3)	12 (8.7)	0 (0.00)	7 (2.9)	3 (0.6)	3 (3.7)
*Follow-up at 5 years of age, N (%)*											
VPT	210 (38.3)	100 (47.6)	96 (91.4)	590 (66.8)	201 (40.9)	520 (65.7)	80 (31.4)	137 (68.8)	320 (66.8)	310 (25.3)	102 (55.1)
EPT	70 (34.5)	52 (36.9)	38 (79.2)	189 (44.6)	79 (29.7)	173 (50.6)	75 (54.3)	52 (44.4)	113 (46.1)	138 (26.7)	42 (51.2)

^a^28–31 completed weeks.

^b^22–27 completed weeks.

Table 2 presents mean costs by cost category and by country. The results show that there was considerable heterogeneity in economic costs by resource category across the countries participating in the study. From a societal perspective, Poland showed the highest total mean cost (€4380) followed by Sweden (€3738). The Netherlands showed the lowest total mean cost (€508), followed by Germany (€852). The table also shows that indirect costs (based on valuations of work absences) were the main driver of economic costs associated with EPT/VPT birth during the fifth year of life. It can be seen from Table 2 that for the majority of the countries, indirect costs accounted for more than half of the mean total societal cost. For instance, in Belgium, mean indirect costs were estimated at €587, which accounted for approximately 58% of the mean total societal cost. In the Swedish cohort, mean indirect costs were €955, which approximated to 26% of the mean total societal cost. Mean costs by preterm birth status in each country are presented in a disaggregated manner for each resource category in Appendix 3. Appendix 4 presents summary statistics for resource use items, as well as the unit costs for each resource input, by country.

**Table 2:** Mean costs during fifth year of life by cost category and by country; mean (SE) (€, 2016 prices).

			Belgium	Denmark	Estonia	France	Germany	Italy	Netherlands	Poland	Portugal	UK	Sweden
Direct medical costs	Hospital outpatient services	Paediatrician	17.29 (2.71)	11.57 (2.80)	18.39 (3.88)	13.88 (0.93)	23.04 (2.05)	52.84 (4.52)	5.24 (1.27)	273.07 (30.34)	34.13 (3.07)	58.48 (9.95)	106.10 (24.26)
		A&E	1.80 (0.30)	8.78 (2.08)	9.19 (2.04)	37.24 (4.46)	32.74 (5.17)	17.26 (1.66)	16.05 (3.36)	5.87 (1.01)	46.90 (3.49)	32.68 (5.75)	72.05 (15.11)
		Neurologist	4.81 (1.05)	1.42 (0.56)	26.11 (18.45)	5.58 (3.13)	2.50 (0.84)	4.09 (0.54)	1.57 (0.91)	30.76 (5.99)	3.90 (0.82)	0.22 (0.05)	32.61 (8.99)
		Ear, nose and throat (ENT)	5.55 (0.73)	21.55 (3.25)	8.94 (1.83)	8.97 (1.36)	7.75 (0.93)	7.59 (1.09)	8.47 (1.78)	18.71 (3.74)	18.81 (1.79)	5.21 (0.70)	33.15 (7.62)
		Ophthalmologist	6.78 (2.01)	9.62 (1.97)	15.26 (2.42)	23.53 (2.64)	24.32 (2.16)	14.43 (1.92)	18.50 (7.20)	29.54 (4.47)	19.85 (2.37)	5.29 (0.51)	65.68 (15.51)
		Speech therapist	111.23 (25.45)	16.56 (5.77)	190.55 (43.48)	200.79 (22.06)	89.49 (12.58)	46.88 (5.41)	52.07 (14.51)	596.64 (102.30)	52.44 (7.81)	29.50 (8.62)	164.18 (68.02)
		Psychologist	9.71 (3.45)	7.66 (3.31)	92.57 (41.37)	22.75 (4.24)	10.47 (8.19)	7.45 (2.08)	1.62 (1.10)	244.65 (72.02)	16.66 (3.94)	2.59 (1.45)	39.20 (14.20)
		Psychiatrist	0.63 (0.25)	0.00 (0.00)	0.55 (0.41)	17.83 (7.79)	0.72 (0.51)	2.67 (0.68)	0.85 (0.67)	2.78 (1.66)	1.50 (0.41)	12.19 (9.35)	39.84 (21.03)
		Physiotherapist	76.46 (13.57)	52.28 (23.09)	71.28 (22.42)	105.92 (13.90)	25.73 (6.51)	72.00 (8.59)	37.95 (12.63)	451.52 (102.28)	173.12 (43.52)	51.95 (16.22)	47.73 (20.70)
		Respiratory specialist	2.30 (0.65)	1.96 (0.85)	6.62 (1.45)	4.88 (0.74)	0.75 (0.22)	3.07 (0.44)	2.82 (1.10)	120.04 (19.84)	22.60 (13.85)	4.37 (2.21)	8.58 (1.83)
		Total hospital outpatient services	236.57 (35.46)	131.38 (28.21)	439.46 (80.94)	441.37 (35.27)	217.51 (22.04)	228.28 (16.72)	145.12 (28.12)	1773.58 (253.39)	389.91 (51.48)	202.49 (38.89)	609.11 (118.88)
	Hospital inpatient services		71.54 (17.82)	49.54 (37.10)	29.37 (10.08)	120.55 (21.01)	254.79 (54.47)	683.28 (127.62)	58.15 (18.95)	78.75 (17.88)	66.79 (17.65)	34.14 (9.33)	202.85 (72.40)
	Community health and social services	GP	24.10 (2.13)	31.74 (3.69)	18.44 (2.16)	39.73 (2.97)	7.99 (1.51)	3.48 (0.61)	16.55 (1.97)	148.38 (20.04)	12.66 (1.30)	25.33 (2.25)	34.37 (4.70)
		Dietician	0.44 (0.25)	1.71 (0.75)	0.55 (0.41)	0.19 (0.19)	0.39 (0.17)	0.77 (0.29)	0.93 (0.47)	1.32 (0.70)	1.09 (0.31)	3.60 (0.83)	11.11 (3.32)
		Health visitor	0.16 (0.10)	2.28 (0.85)	14.51 (4.44)	0.00 (0.00)	0.78 (0.39)	7.24 (5.39)	1.49 (1.11)	2.81 (1.28)	6.50 (3.45)	2.26 (0.72)	0.00 (0.00)
		School nurse	4.86 (1.84)	0.00 (0.00)	0.67 (0.26)	0.00 (0.00)	0.09 (0.06)	0.01 (0.01)	3.60 (0.65)	1.98 (0.57)	1.54 (0.43)	6.96 (1.59)	0.00 (0.00)
		Physiotherapist	37.71 (13.79)	13.20 (5.75)	10.59 (4.56)	29.19 (20.19)	99.22 (15.28)	1.46 (0.75)	2.87 (1.77)	309.51 (81.45)	34.22 (6.45)	47.99 (20.68)	135.62 (99.96)
		Early childhood intervention	15.28 (9.37)	23.92 (20.13)	11.71 (10.95)	0.00 (0.00)	21.15 (5.06)	16.88 (5.04)	0.52 (0.52)	243.59 (91.02)	100.25 (17.59)	886.91 (416.92)	60.28 (19.01)
		Others	1.83 (0.32)	2.32 (0.66)	12.99 (1.75)	0.47 (0.14)	3.69 (0.48)	0.00 (0.00)	9.10 (0.98)	46.88 (4.39)	6.61 (0.79)	1.52 (0.30)	108.17 (14.88)
		Total community health and social services	84.38 (17.66)	75.16 (21.34)	69.45 (13.18)	69.58 (20.40)	133.32 (17.42)	29.84 (7.62)	35.06 (3.45)	754.47 (155.87)	162.86 (23.67)	974.57 (424.11)	349.55 (106.27)
Direct non-medical costs	Equipment costs^a^		16.48 (7.77)	142.07 (48.09)	29.10 (12.18)	14.79 (2.82)	7.57 (2.40)	23.82 (10.17)	6.90 (2.96)	207.28 (73.51)	18.87 (4.43)	12.40 (7.70)	202.25 (180.00)
	Out-of-pocket family expenses		23.52 (8.09)	61.97 (19.23)	172.80 (103.13)	20.09 (6.36)	15.55 (4.80)	162.52 (37.49)	9.75 (5.64)	729.97 (164.84)	84.49 (19.02)	28.48 (17.81)	1419.72 (851.16)
Indirect costs	Valuation of time off work		587.10 (165.41)	729.22 (314.42)	732.75 (148.67)	220.53 (48.17)	223.18 (80.46)	617.19 (111.02)	252.87 (174.50)	835.54 (239.52)	734.35 (149.80)	302.42 (91.20)	954.93 (423.13)
Total societal costs^b^			1019.59 (192.66)	1189.33 (337.22)	1472.92 (219.72)	886.91 (78.14)	851.92 (113.95)	1744.93 (201.10)	507.85 (197.22)	4379.59 (604.41)	1457.28 (205.51)	1554.51 (500.28)	3738.41 (1122.62)

*GP* general practitioner, *A&E* accident and emergency.

^a^These costs include any special equipment related to infants such as wheelchair, brace, hearing aids, or shoes.

^b^Sum of all the cost categories.

Table 3 shows the result of the country-specific GLM regressions using two comparators for gestational age status (VPT vs EPT). These models control for clinical and sociodemographic factors expected to have influenced the dependent variable, namely total societal cost during the fifth year of life. It was generally observed that lower gestational age at birth was associated with higher economic costs. All countries showed elevated economic costs for children born at < 28 weeks except for Belgium, Estonia and Germany. Higher costs were reported for the EPT group for eight countries (Denmark, France, Italy, Netherlands, Poland, Portugal, UK and Sweden). Separate regression results based on four categories of gestational age (< 26, 26–27, 28–29, 30–31 weeks) are presented in Appendix 5. The appendix shows a similar pattern in terms of the direction of signs for the cost coefficients.

**Table 3:** Multivariate analysis to examine the association between total societal costs at 5 years of age and gestational age at birth (generalised linear model); gestational age presented in two categories (€, 2016 prices).

		Belgium	Denmark	Estonia	France	Germany	Italy	Netherlands	Poland	Portugal	UK	Sweden
Gestational age (ref. 28–31 weeks)	extremely preterm (22–27 weeks)	−1175.41	2213.02**	−536.64	967.53***	−59.06	1004.04**	66.10	4723.60*	1763.38***	5784.87***	1866.26
		(617.90)	(849.17)	(394.72)	(219.40)	(733.48)	(379.66)	(360.87)	(1850.20)	(483.18)	(1554.24)	(1321.00)
SGA (ref >10th centile)	≤10th centile	3358.42**	117.32	−123.15	285.67	1003.79	−47.89	494.65	−1569.73	1579.45***	1414.83	1372.68
		(1294.46)	(627.21)	(361.43)	(164.41)	(613.87)	(276.11)	(396.86)	(1515.57)	(410.62)	(1205.84)	(1397.27)
BPD (ref: none)	Yes	3466.09	−1033.35	−313.68	936.12**	4872.83*	2772.25**	329.78	6161.45*	910.73	−1535.90	−494.35
		(1827.91)	(672.04)	(529.24)	(319.98)	(2303.47)	(928.19)	(437.65)	(2846.83)	(597.60)	(1137.82)	(1393.71)
Severe congenital anomalies (ref: none)	Yes	20,706.97	−1937.11***	2293.96	400.48	2809.61	5602.29***	167.95	6127.73***	−587.17	−1640.43	7014.01
		(14,772.26)	(480.04)	(1948.65)	(305.64)	(1835.50)	(1449.55)	(529.63)	(1844.88)	(540.26)	(1297.84)	(3710.12)
Any morbidity (ref: none)	Yes	2641.16	2868.84	794.83	506.01	3334.91	3056.29***	−501.63	5033.25**	3299.61***	9950.68***	14025.99*
		(1808.76)	(2741.21)	(768.57)	(375.98)	(1937.52)	(755.07)	(393.79)	(1808.53)	(937.88)	(2704.98)	(6514.29)
Sex (ref: female)	Male	382.69	2978.37**	1290.53***	504.64***	736.63	497.81	1765.72***	3977.92**	1013.32***	7180.26***	220.76
		(512.83)	(915.96)	(342.57)	(140.35)	(592.99)	(260.89)	(430.14)	(1364.79)	(303.01)	(1185.80)	(1039.24)
One or both parents unemployed (ref: employed)	Unemployed	14,432.55	10,734.50	2423.82	−470.35**	−43.10	−1068.57**	−882.83*	−3656.24	3995.06***	976.20	6819.47
		(14,244.35)	(10,729.84)	(1409.20)	(159.83)	(3179.03)	(326.04)	(402.09)	(1969.21)	(851.86)	(2103.27)	(7394.32)
Maternal education (ref: higher education)	High school or less	−897.93	1117.39	−73.61	−251.69	1218.37*	1711.91***	628.66*	−2620.07	390.22	−576.10	4227.19**
		(560.78)	(685.33)	(349.12)	(158.78)	(604.74)	(275.68)	(275.02)	(1574.33)	(294.14)	(1000.55)	(1447.61)
Country of birth for mothers (ref: native)	European born	−2654.23***	2551.86	5242.38	1593.64	−996.01	−158.98	−1603.83***		−1701.30***		−1736.61
		(546.51)	(3271.53)	(7656.64)	(1014.58)	(712.32)	(371.94)	(415.99)		(349.96)		(1547.48)
	Non-European born	−2338.90***	−1440.20		−112.63	2146.35	−649.65	−1053.81***		−167.04	−5227.31***	−3277.42**
		(686.74)	(976.38)		(146.82)	(1892.65)	(421.22)	(315.16)		(534.79)	(796.11)	(1030.97)
Maternal age (ref. 25–34)	<25	−1916.05***	-410.85	−830.70*	301.03	1408.48	−1157.65***	6368.88*	3064.41	−193.89	11582.96***	−4047.43***
		(539.59)	(562.48)	(415.56)	(241.53)	(1528.82)	(232.47)	(3011.20)	(1799.24)	(500.63)	(3127.62)	(939.58)
	>34	2511.61	2874.28*	−310.69	88.78	907.71	1690.84***	105.19	7080.21**	−451.09	3016.95**	537.82
		(1439.87)	(1143.14)	(403.20)	(176.96)	(797.51)	(333.83)	(260.27)	(2317.43)	(319.88)	(1047.54)	(1165.99)
First child (ref: not the first child)		−28.85	166.84	71.66	−30.17	1350.67*	−210.54	−1171.82	950.62	570.08*	−6508.81***	1536.53
		(512.54)	(613.82)	(327.69)	(141.60)	(587.24)	(292.08)	(640.74)	(1486.52)	(283.13)	(1471.91)	(1108.16)
Multiple (ref: singleton)	Twins	−753.33	−1253.49	−550.47	−231.87	442.82	−1347.15***	−573.35	3906.34*	−271.95	-4395.66***	−1919.01*
		(505.37)	(752.89)	(409.75)	(139.43)	(764.40)	(248.84)	(306.23)	(1738.78)	(324.70)	(758.52)	(931.50)
	Triplets or quadruplets		−1865.22*	−1534.42***	740.97	−432.34	39.90	−1323.68*	−1824.70	37.38	−4383.36***	680.42
			(788.76)	(250.53)	(1009.24)	(974.63)	(701.51)	(526.42)	(5691.99)	(1085.50)	(972.75)	(3600.21)

Morbidity: intraventricular haemorrhage grades III–IV (IVH), periventricular leukomalacia (PVL), retinopathy of prematurity stages III–V (ROP), or necrotising enterocolitis needing surgery (NEC).

*SGA* small for gestational age, *BPD* bronchopulmonary dysplasia.

*p < 0.05.

**p < 0.01.

***p < 0.001.

The results of the multi-level analyses using either total health and social service costs or total societal costs as the dependent variable are given in Table 4. These are broadly in line with the results of Table 3. For the model using total societal costs as the dependent variable, gestational age at birth of <26, 26–27 and 28–29 weeks was associated with higher costs than the reference group by €2756 (*p* < 0.001), €752 (*p* < 0.01) and €657 (*p* < 0.01), respectively. The presence of a congenital anomaly, neonatal morbidity, male and low maternal education status were associated with significantly higher costs compared to their referent groups by €3113 (*p* < 0.001), €3234 (*p* < 0.001), €1227 (*p* < 0.001) and €625 (*p* < 0.01), respectively. When total health and social care services cost was used as the dependent variable, the signs of coefficients showed similar patterns to the results for total societal costs.

**Table 4:** Multi-level analysis to examine the association between total costs (€, 2016 prices) at 5 years of age and gestational age at birth (generalised linear model)^a,b,c^.

			Sensitivity analyses excluding diagnosis of BPD and any neonatal morbidity		Sensitivity analyses excluding diagnosis of BPD, any neonatal morbidity and presence of a congenital anomaly	
	Total health and social care services costs per child^d^	Total societal costs per child	Total health and social care services costs per child^d^	Total societal costs per child	Total health and social care services costs per child^d^	Total societal costs per child
*Perinatal characteristics*						
Gestational age (ref. 30–31 weeks)						
<26	1920.63***	2755.98***	2908.22***	4577.41***	2858.57***	4236.51***
	(520.31)	(707.71)	(742.44)	(983.53)	(760.86)	(994.12)
26–27	490.93**	752.34**	789.06***	1427.76***	876.90***	1514.57***
	(170.03)	(282.32)	(216.11)	(343.76)	(243.89)	(383.60)
28–29	418.67**	657.09**	525.08***	832.38***	471.20**	801.29**
	(136.14)	(233.56)	(150.83)	(237.61)	(146.40)	(249.53)
SGA (ref >10th centile)						
≤10th centile	212.29	307.9	243.35*	349.04	320.69*	420.35
	(125.01)	(228.03)	(123.98)	(226.75)	(136.41)	(238.81)
BPD (ref: none)						
Yes	788.94**	1128.05**				
	(261.05)	(408.98)				
Severe congenital anomalies (ref: none)						
Yes	1257.74**	3112.72***	1245.99**	3093.42***		
	(389.97)	(773.21)	(393.11)	(783.60)		
Any morbidity (ref: none)						
Yes	1467.31***	3233.58***				
	(395.47)	(697.97)				
Sex (ref: female)						
Male	674.37***	1226.62***	536.18***	1195.17***	548.00***	1126.70***
	(164.96)	(243.35)	(147.37)	(249.51)	(155.33)	(264.46)
*Sociodemographic characteristics*						
Household employment status (ref: employed)						
At least one parent unemployed	239.81	426.28	253.00	1047.42*	235.13	1082.34*
	(197.78)	(378.60)	(194.76)	(454.73)	(195.44)	(478.32)
Maternal education (ref: higher education)						
High school or less	256.48*	625.17**	374.41**	744.56***	360.95**	666.95**
	(119.16)	(218.60)	(129.07)	(225.94)	(131.32)	(232.67)
Country of birth for mothers (ref: native)						
European born	79.75	−477.84	−276.23	−669.16	−364.99	−911.15*
	(279.06)	(428.14)	(216.82)	(384.38)	(216.71)	(379.55)
Non-European born	−552.79***	−577.29*	−574.66***	−431.75	−622.14***	−543.01
	(162.64)	(282.16)	(165.87)	(293.31)	(177.83)	(299.58)
Mother age at childbirth (ref. 25–34 years)						
<25	260.95	493.15	259.29	729.02	299.15	824.27
	(195.35)	(368.39)	(190.96)	(393.84)	(201.54)	(421.28)
>34	303.08*	671.45**	223.50	538.50*	195.46	451.82
	(139.89)	(259.00)	(126.80)	(244.42)	(126.19)	(246.18)
Parity (ref: multiparous)						
	−95.08	−52.68	−143.38	−41.63	−181.20	−46.74
	(113.69)	(211.56)	(113.06)	(207.83)	(118.25)	(213.47)
Multiple (ref: singleton)						
Twins	−96.95	−383.66	−26.26	−192.33	−99.09	−369.79
	(111.53)	(213.93)	(108.68)	(217.18)	(109.18)	(221.39)
Triplets or quadruplets	222.33	−809.38	179.31	−801.25	390.44	−600.09
	(341.46)	(439.62)	(321.05)	(430.76)	(375.19)	(484.86)

Standard errors in parentheses.

*SGA* small for gestational age, *BPD* bronchopulmonary dysplasia.

Morbidity: intraventricular haemorrhage grades III–IV (IVH), periventricular leukomalacia (PVL), retinopathy of prematurity stages III–V (ROP), or necrotising enterocolitis needing surgery (NEC).

*p < 0.05.

**p < 0.01.

***p < 0.001.

^a^Variance of the intercept in the multi-level model is 0.07.

^b^*p* value for LR test is <0.001 for 1) and 2), which means there is significant difference between a single-level model and multi-level model.

^c^Negative binomial family with log link was used for this GLM.

^d^This variable excludes expenses incurred by parents and valuation of work absences.

In the sensitivity analyses that excluded diagnosis of BPD, any neonatal morbidity and presence of a congenital anomaly, the results were similar to the results of the baseline model in terms of statistical significance and the signs of coefficients. Incremental societal costs were estimated at €4237 (*p* < 0.001), €1515 (*p* < 0.01) and €801 (*p* < 0.01), for children born at <26, 26–27 and 28–29 weeks, respectively, in comparison to the reference group born at 30–31 weeks. Incremental societal costs were elevated by €1482, €763 and €144 at <26, 26–27 and 28–29 weeks, respectively, in comparison to the baseline model. The results of the model that excluded diagnosis of BPD, any neonatal morbidity and presence of a congenital anomaly reduced incremental costs by €341, €-87 and €31 at <26, 26–27 and 28–29 weeks, respectively, in comparison to the model that only excluded diagnosis of BPD and any neonatal morbidity. The results of the further sensitivity analysis that additionally excluded the employment status variable are reported in Appendix 6 and revealed a broadly similar pattern of cost coefficients for the gestational age variables. Inverse probability weight-adjusted regression analysis was separately performed to account for attrition, and the results are presented in Appendix 7.

## Discussion

This study reports the economic costs associated with EPT and VPT birth at 5 years of age in 11 European countries using data from a large cohort of EPT and VPT children. This is, to the best of our knowledge, the first comparative study of the economic costs associated with care for children born EPT and VPT across European countries.

This study shows a number of findings. Firstly, it confirms that economic costs are negatively associated with gestational age at birth, with children born at <26, 26–27 and 28–29 weeks generally incurring higher societal costs than children born at 30–31 weeks even during the fifth year of life. This agrees with the conclusions of the study by Mangham et al.^17^ In that particular study,^17^ the authors entered live birth and gestational age data available for England and Wales into a decision-analytic model to calculate the mean incremental cost per preterm child surviving to 18 years. The authors found the public sector cost per preterm child surviving to 18 years increased by degree of prematurity, from £103,831 for VPT children to £136,790 for EPT children (2006 prices). The authors also found that, compared with a term child surviving to 18 years, the incremental cost per preterm child surviving to this age was £22,764.

Secondly, our multi-level analysis shows that most of the clinical and sociodemographic variables selected for inclusion are predictors of economic costs during the fifth year of life. With the exception of SGA status, household employment status, maternal country of birth, parity and multiplicity, all other variables were significant predictors of economic costs. Our multi-level analysis also found that male children incurred higher costs than female children. This is in line with research showing increased risks of long-term neurological and motor impairments after preterm birth in male children.^35^ A number of studies have revealed an association between parents’ socioeconomic status and preterm birth^2,36,37^ mediated through income and education status, and our results showing increased costs associated with low maternal education and unemployed status are consistent with this literature.^38^ The only covariate with potentially contrasting results to the existing literature is multiplicity, as singletons were found to incur higher costs than individual twins (but not sets of twins) in our analysis, but this estimate was not statistically significant.

A key driver of increased economic costs for children born VPT was indirect costs. One study performed in Finland reported first year parental wage losses of €5990 (1997 prices) for children with birth weights of <1000 g, whereas the loss was €880 for controls, although the study did not categorise the children by gestational age. These costs increased to €8175 in the second year for the parents of children born with low birth weights.^39^ Clearly, further effort should be placed on developing granulated approaches to estimating indirect costs associated with preterm birth and development.

Although a distinct, dominating pattern is not found in this study, the results suggest that high costs are incurred for VPT and EPT born children. Male children had higher costs than female children in all of the participating countries, despite effects not being statistically significant in all countries. Likewise, the multi-level model showed that maternal age at delivery above 34 years was associated with higher costs.

A strength of our study lies in the comprehensive estimation of costs associated with EPT/VPT birth across several European countries with differing care systems.

The study population was drawn from 11 countries, offering a large sample of approximately 3700 EPT/VPT born children. In addition, we constructed a database of unit costs and economic costs for ten categories of hospital outpatient services, seven categories of community health and social services, equipment costs and indirect costs across 11 European countries. Another strength of this study is the representativeness of the cohort as the regions were selected to represent the diversity of organisational systems in European countries and in accordance with their availability to carry out the study. The preterm birth rates in this study resemble those in the EURO-Peristat report.^40^ The EPICE cohort^22^ shows preterm birth rates ranging from 0.9 to 1.5% for the regions in participating countries while the EURO-Peristat report^40^ shows preterm birth rates ranging from 0.9 to 1.3% (Appendix 8). Prevalence rates in the study sample were similar to those quoted for England by Brilleman et al.^41^ Loss to follow-up in the EPICE cohort on which the SHIPS cohort was built has been associated with mainly sociodemographic and some perinatal characteristics.^22^ Non-responders are more likely to be children of younger, foreign-born mothers with lower educational levels.

The multi-level generalised linear modelling that we applied is an appropriate analytical method for pooling results of within-country cost estimates. Multi-level analysis is suggested for costing or cost-effectiveness analysis at the country level as a means for pooling cost data across countries.^42^ Although the intra-class correlation (ICC) is an indicator examining whether multi-level analysis is appropriate by calculating the ratio between the variance of the intercept at multi-level and the sum of the variance of the intercept and the variance of residuals, the ICC could not be estimated for the negative binomial model that was used for this study because the negative binomial and Poisson models assume that the variance of the error term is a function of the predicted values of parameters.

Our study design has caveats that should be borne in mind by readers. First, our estimates of economic costs are compound values informed by parental-reported resource use values and expenses over the fifth year of their child’s life. Previous research has indicated that parents accurately recall their child’s hospital service utilisation over extended periods when validated against medical records, but tend to under-report their child’s use of other services.^43^ If this were the case for our study, our absolute costs for community service utilisation might be underestimates. Likewise, there can be recall biases when providing information about lost working days due to a child’s health status. For twins and triplets, we attempted to minimise errors by tailoring the study introductory letters and research instruments to the children of focus so that the parents could complete each set of questions with a particular named child in mind.

Second, there is considerable heterogeneity around the values for resource use across countries. This might be related, at least in part, to differing health and social care practices and systems across countries. For example, some countries do not offer a ‘school nurse’ system. As a result, the mean number of school nurse contacts will differ significantly between countries. However, this is not a unique problem for this study, and it is not unexpected. Another study performed in Europe regarding dental costs reported that heterogeneity in economic costs across countries was partly attributable to the different systems and types of services offered by countries.^44^ Third, the accuracy of the estimated costs relies on the availability of unit costs estimated in accordance with opportunity cost principles. Although we endeavoured to collect appropriate unit cost data for each country, central databases listing relevant unit costs are not available in every country. To mitigate this, we consulted with local health economists and researchers, and we referred to relevant literature. Moreover, we attempted to minimise differences in living standards between the countries by applying PPPs. As costing analysis for multi-country comparisons is intrinsically open to the issue of heterogeneity in prices for services, it is important to minimise the differences across countries. This is to adjust for differences in purchasing power and allow for comparisons of living standards and economic productivity between countries.

Fourth, even though the analysis was conducted from a stated societal perspective, we recognise that some potentially important resource/cost categories may have been excluded. For example, the potential effects of VPT birth on long-term reductions in parental employment prospects were not captured. Nevertheless, this is common in many economic analyses with stated societal perspectives. For example, many economic analyses exclude values associated with use of vacation times^45^ and replacement of labour time.^46^

Fifth and last, our model specification used for multi-level mixed effects GLMs incorporated several confounders. An alternative analytical framework is offered by mediation analysis to capture the indirect effect of variables plausibly lying on the causal pathway between gestational age at birth and economic outcomes.

It is very difficult to pinpoint the reasons for cost variations and to generalise these across countries with any degree of certainty without being overly speculative. Economic cost estimates calculated in our study combine use of multiple types of services/resources with unit costs (prices) for these services. Thus, at the very top level, variations across countries are compounded by variation in resource use and variation in unit costs. While health systems across European countries are broadly uniform in relation to provision of care, cross-country variations in resource use are likely to be caused by subtler structural characteristics of the systems including availability of services, ease of access in the different enrolled regions of the participating countries as well as wider predisposing factors. Unit costs also vary across countries, for a host of reasons including differences in health care professionals’ salaries and cost of equipment. In relation to direct non-medical costs borne by a child’s parents and family, variation in our costs estimates may, similarly, be due to heterogeneity in the type, use and unit cost of equipment, the type, cost and reimbursement level of other expenses such as house alterations.

Our findings can be used to inform clinical and budgetary service planning and provide policy-relevant information for cross-country, longitudinal and other cost comparisons. Clinical and budgetary planning of services for preterm born children can be informed by estimates of resource use and economic costs through stages of childhood.^47^ Furthermore, the information provided in this study can act as cost inputs, when combined with cost estimates for other ages, in cost-effectiveness analyses of interventions that delay birth for infants who otherwise would have been born at gestations of 26–31 weeks.^17^ In economic evaluations of preventive or treatment interventions based on decision-analytic models, our data can be used to value health states during the fifth year of life in monetary terms, where the probability of survival up to 5 years of age is factored in elsewhere in the models. This study has generated a body of data that can act as inputs into future economic models, and allows the reader to assess the suitability of the evidence for their particular context.

For policy makers, this study adds to sparse evidence about the main cost drivers associated with preterm birth in European countries. The full effects of impairment associated with EPT/VPT birth on economic outcomes clarify only with time. Cohort studies with longer-term follow-up into adolescence and adulthood and whole-country record linkage studies are needed to ascertain long-term economic outcomes such as long-term use of health and social care services, employment and occupational status, income, receipt of social welfare benefits and reproductive health, which might in turn have economic sequelae.

## Supplementary information

Supplementary information
