# Supplementary material for: Economic costs at age five associated with very preterm birth: multinational European cohort study
Source: Pediatr Res. 2021 Nov 12;92(3):700–11. doi: 10.1038/s41390-021-01769-z (PMC9556316; doi:10.1038/s41390-021-01769-z)
Supplement: Supplementary file 1 — Supplementary information [file 41390_2021_1769_MOESM1_ESM.docx]

# Appendices

**Appendix 1: Sources for unit costs of resource use items**

| **Country** | **Sources of unit costs** |
| --- | --- |
| Belgium | Local PI’s advice , Relevant literature [46-50] |
| Denmark | Kok et al. [51], Relevant literature [52-54] |
| Estonia | Results and costs of health care of very preterm infants in Estonia [55], Relevant literature [56] |
| France | Relevant literature  [48, 56-62] |
| Germany | Relevant literature [48, 58, 63-67] |
| Italy | Nomenclatore specialistica 2013 [68], Relevant literature [69, 70] |
| Netherlands | Relevant literature [47, 48, 71-76] |
| Poland | [56, 77-82] |
| Portugal | Price consultation in NHS [83], Relevant literature [56] |
| Sweden | Relevant literature [48, 56, 66, 84-87] |
| UK | PSSRU [88], Relevant literature [89-93] |
|  |  |

**Appendix 2: A flow chart of participants, by preterm birth status**


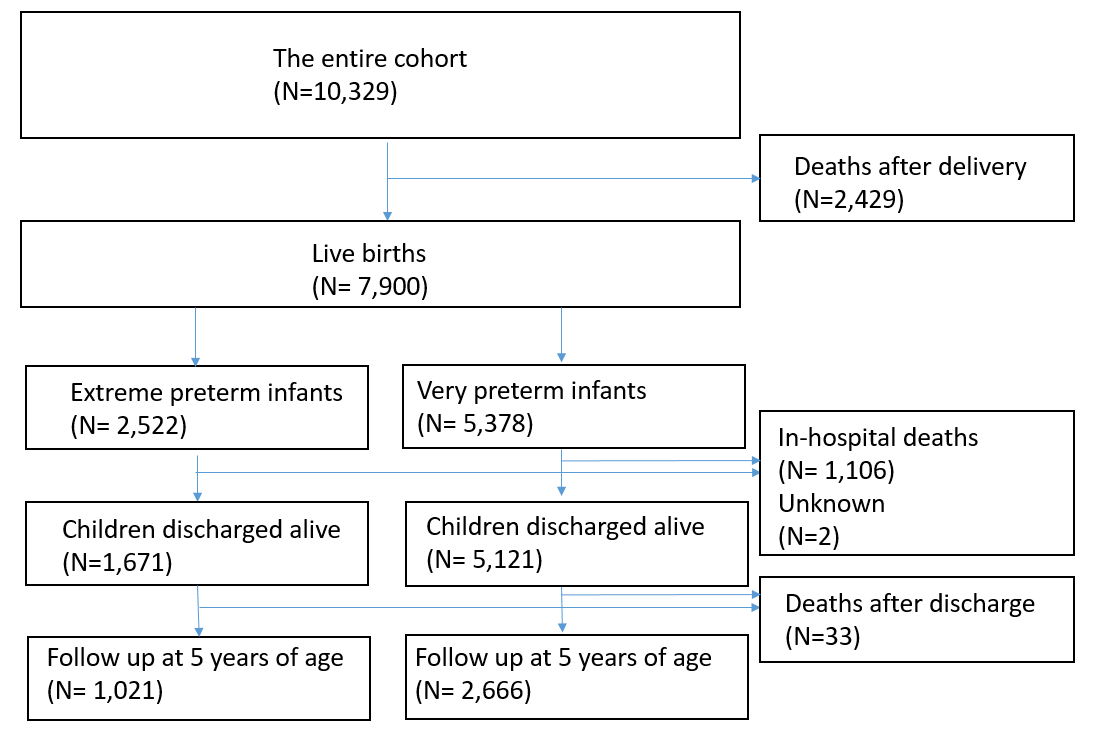


**Appendix 3: Costs for each resource category in each country by preterm birth status, mean (SE) (€, 2016 prices)**

|  |  | **Belgium** | | **Denmark** | | **Estonia** | | **France** | | **Germany** | | **Italy** | |
| --- | --- | --- | --- | --- | --- | --- | --- | --- | --- | --- | --- | --- | --- |
|  |  | **VPT** | **EPT** | **VPT** | **EPT** | **VPT** | **EPT** | **VPT** | **EPT** | **VPT** | **EPT** | **VPT** | **EPT** |
| **Hospital outpatient services** | **Paediatrician** | 13.59 (1.81) | 27.33  (8.76) | 12.64 (4.39) | 9.97  (2.40) | 21.16 (5.19) | 12.31 (4.88) | 14.77 (1.02) | 12.04 (1.94) | 26.14 (2.51) | 17.30 (3.50) | 49.34 (2.93) | 60.95 (13.38) |
|  | **A&E** | 1.29  (0.28) | 3.19  (0.79) | 7.02  (2.59) | 11.40  (3.44) | 9.24  (2.15) | 9.06 (4.54) | 42.04 (6.00) | 27.25 (5.74) | 35.63 (7.29) | 27.41 (5.92) | 18.32 (2.02) | 14.81 (2.89) |
|  | **Neurologist** | 5.47  (1.34) | 3.03  (1.37) | 1.19  (0.59) | 1.77  (1.08) | 7.50  (2.26) | 66.81 (58.59) | 6.54  (4.55) | 3.57  (1.84) | 2.04  (0.62) | 3.36  (2.10) | 3.73  (0.66) | 4.93  (0.96) |
|  | **Ear, Nose and Throat (ENT)** | 5.29  (0.82) | 6.26  (1.58) | 20.24 (3.48) | 23.49  (6.21) | 10.92 (2.54) | 4.62  (1.70) | 9.89  (1.93) | 7.05  (1.19) | 8.82  (1.27) | 5.77  (1.21) | 7.34  (0.73) | 8.17  (3.20) |
|  | **Ophthalmologist** | 7.33  (2.71) | 5.30  (1.35) | 8.04  (2.14) | 11.97  (3.72) | 15.27 (2.64) | 15.24 (5.16) | 25.43 (3.30) | 19.59 (4.37) | 24.70 (2.76) | 23.62 (3.46) | 15.53 (2.64) | 11.88 (1.78) |
|  | **Speech therapist** | 112.35 (32.65) | 108.22 (33.15) | 14.47 (4.81) | 19.67 (12.48) | 193.29 (51.82) | 184.58 (80.54) | 185.60 (25.13) | 232.43 (43.40) | 84.09 (13.11) | 99.49 (26.42) | 40.35 (5.92) | 62.02 (11.57) |
|  | **Psychologist** | 11.84 (4.69) | 3.94  (1.69) | 3.57  (1.67) | 13.74  (7.83) | 83.73 (56.35) | 111.93 (47.53) | 20.33 (5.20) | 27.79 (7.33) | 3.25  (1.41) | 23.83 (23.21) | 7.72  (2.52) | 6.82  (3.66) |
|  | **Psychiatrist** | 0.17  (0.12) | 1.86  (0.86) | 0.00  (0.00) | 0.00  (0.00) | 0.80  (0.60) | 0.00  (0.00) | 20.03 (11.19) | 13.24 (5.83) | 0.27  (0.14) | 1.57  (1.43) | 3.06  (0.95) | 1.77  (0.54) |
|  | **Physio therapist** | 69.50 (16.37) | 95.29 (23.82) | 56.15 (29.07) | 46.51 (37.93) | 59.60 (23.43) | 96.83 (50.04) | 84.78 (14.19) | 149.95 (30.95) | 31.20 (9.34) | 15.60 (6.79) | 58.42 (9.49) | 103.44 (18.02) |
|  | **Respiratory or asthma specialist** | 1.22  (0.46) | 5.23  (2.07) | 1.79  (1.11) | 2.22  (1.32) | 6.70  (1.64) | 6.45  (2.93) | 4.48  (0.78) | 5.72  (1.59) | 1.10  (0.33) | 0.10  (0.10) | 3.03  (0.52) | 3.17  (0.87) |
| **Total Hospital outpatient services** | **Mean (SE)** | 228.04 (44.81) | 259.63 (50.84) | 125.10 (30.81) | 140.75 (53.30) | 408.21 (95.46) | 507.82 (152.60) | 413.88 (39.50) | 498.61 (71.08) | 217.22 (23.91) | 218.04 (44.66) | 206.84 (17.46) | 277.94 (37.86) |
|  | **Median (IQR)** | 0.00  (0.00) | 0.00  (20.2) | 0.00  (125.0) | 0.00  (125.0) | 33.5 (387.1) | 61.6 (247.2) | 0.00 (126.5) | 22.7 (189.8) | 0.00  (56.8) | 0.00  (155.8) | 0.00  (162.8) | 67.7 (166.3) |
| **Hospital inpatient services** | **In hospital days** | 57.90 (20.10) | 108.41 (37.42) | 74.08 (61.72) | 12.98 (9.15) | 22.22 (9.29) | 45.01 (24.95) | 113.80 (22.69) | 134.59 (44.33) | 287.77 (78.30) | 193.81 (55.91) | 581.71 (128.90) | 918.49 (299.93) |
|  | **Median (IQR)** | 0.00  (0.00) | 0.00  (0.00) | 0.00  (0.00) | 0.00  (0.00) | 0.00  (0.00) | 0.00  (0.00) | 0.00  (0.00) | 0.00  (0.00) | 0.00  (0.00) | 0.00  (0.00) | 0.00  (0.00) | 0.00  (0.00) |
| **Community health and social services** | **GP** | 24.95 (2.54) | 21.80  (3.91) | 32.72 (4.83) | 30.28  (5.74) | 19.67 (2.83) | 15.75 (3.04) | 39.12 (2.35) | 41.01 (7.75) | 7.88  (1.61) | 8.21  (3.10) | 3.58  (0.77) | 3.28  (0.93) |
|  | **Dietician** | 0.25  (0.25) | 0.97  (0.65) | 2.10  (1.17) | 1.12  (0.67) | 0.80  (0.60) | 0.00  (0.00) | 0.00  (0.00) | 0.58  (0.58) | 0.47  (0.22) | 0.24  (0.24) | 0.85  (0.40) | 0.61  (0.24) |
|  | **Health visitor** | 0.05  (0.05) | 0.44  (0.33) | 3.24  (1.39) | 0.84  (0.42) | 14.62 (5.65) | 14.26 (6.95) | 0.00  (0.00) | 0.00  (0.00) | 0.50  (0.35) | 1.31  (0.90) | 2.55  (2.35) | 18.10 (17.03) |
|  | **School Nurse** | 3.37  (0.59) | 8.89  (6.61) | 0.00  (0.00) | 0.00  (0.00) | 0.84  (0.37) | 0.29  (0.17) | 0.00  (0.00) | 0.00  (0.00) | 0.07  (0.07) | 0.13  (0.13) | 0.01  (0.01) | 0.00  (0.00) |
|  | **Occupational therapist** | 38.87 (17.35) | 34.56 (20.27) | 16.87 (8.94) | 7.75  (5.25) | 9.94  (5.23) | 12.00 (9.05) | 21.53 (21.14) | 45.14 (44.02) | 82.59 (17.96) | 129.98 (28.10) | 1.19  (0.84) | 2.08  (1.54) |
|  | **Early childhood intervention** | 17.96 (12.76) | 8.04  (3.88) | 1.93  (0.90) | 56.66 (50.06) | 0.32  (0.32) | 36.62 (34.88) | 0.00  (0.00) | 0.00  (0.00) | 19.58 (6.37) | 24.05 (8.33) | 19.86 (6.51) | 9.99  (7.19) |
|  | **Others** | 2.31  (0.42) | 0.52  (0.30) | 2.91  (0.95) | 1.44  (0.83) | 14.33 (2.29) | 10.05 (2.45) | 0.64  (0.20) | 0.12  (0.12) | 3.64  (0.58) | 3.79  (0.85) | 0.00  (0.00) | 0.00  (0.00) |
| **Total Community health and social services** | **Mean (SE)** | 87.77 (22.65) | 75.21 (22.99) | 59.77 (10.98) | 98.08 (50.61) | 60.52 (9.78) | 88.98 (36.25) | 61.29 (21.23) | 86.84 (44.77) | 114.74 (20.19) | 167.70 (32.67) | 28.03 (7.35) | 34.05 (18.68) |
|  | **Median (IQR)** | 0.00  (25.0) | 0.00  (50.0) | 0.00  (52.1) | 0.00  (52.1) | 28.1  (50.0) | 41.1  (63.0) | 0.00  (46.2) | 0.00  (69.4) | 0.00 (0.00) | 0.00  (0.00) | 0.00  (0.00) | 0.00  (0.00) |
| **Equipment costs¹** | **Mean (SE)** | 19.30 (10.52) | 8.87 (4.30) | 134.36 (51.07) | 153.55 (92.69) | 33.89 (17.22) | 18.63 (9.44) | 12.73 (2.18) | 19.09 (7.42) | 9.21  (3.52) | 4.53  (2.05) | 21.40 (12.83) | 29.44 (15.99) |
|  | **Median (IQR)** | 0.00  (0.00) | 0.00  (0.00) | 0.00  (0.00) | 0.00  (0.00) | 0.00  (0.00) | 0.00  (0.00) | 0.00  (0.00) | 0.00  (0.00) | 0.00  (0.00) | 0.00  (0.00) | 0.00  (0.00) | 0.00  (0.00) |
| **Out of pocket expenses** | **Mean (SE)** | 24.12 (10.21) | 21.92 (11.67) | 45.48 (21.00) | 86.52 (36.24) | 249.24 (149.89) | 5.58  (3.89) | 20.06 (8.43) | 20.15 (8.71) | 16.07 (5.50) | 14.61 (9.16) | 153.05 (50.66) | 184.44 (41.16) |
|  | **Median (IQR)** | 0.00  (0.00) | 0.00  (0.00) | 0.00  (0.00) | 0.00  (0.00) | 0.00  (0.00) | 0.00  (0.00) | 0.00  (0.00) | 0.00  (0.00) | 0.00  (0.00) | 0.00  (0.00) | 0.00  (0.00) | 0.00  (0.00) |
| **Valuation of time off work** | **Mean (SE)** | 336.91 (99.33) | 1263.72 (548.94) | 383.69 (258.01) | 1243.83 (681.24) | 793.38 (190.09) | 600.11 (228.89) | 223.06 (37.11) | 215.28 (126.91) | 170.93 (29.19) | 319.81 (222.98) | 509.88 (117.06) | 865.70 (248.82) |
|  | **Median (IQR)** | 0.00  (0.00) | 0.00  (0.00) | 0.00  (0.00) | 0.00  (300.0) | 0.00  (0.00) | 0.00  (0.00) | 0.00  (0.00) | 0.00  (0.00) | 0.00  (0.00) | 0.00  (0.00) | 0.00  (0.00) | 0.00  (0.00) |
| **Total societal costs^2^** | **Mean (SE)** | 754.03 (146.18) | 1737.76 (592.38) | 822.48 (279.10) | 1735.71 (728.67) | 1567.46 (278.22) | 1266.13 (348.77) | 844.82 (77.13) | 974.56 (179.59) | 815.94 (110.96) | 918.49 (251.95) | 1500.90 (208.35) | 2310.07 (459.36) |
|  | **Median (IQR)** | 0.00 (107.7) | 0.00  (125.0) | 0.00  (218.7) | 0.00  (229.1) | 92.4 (1601.8) | 333.1 (1752.1) | 0.00 (246.7) | 69.4 (508.5) | 0.00  (97.2) | 0.00  (354.2) | 0.00  (536.4) | 68.2 (285.2) |

(Continued)

|  |  | **Netherlands** |  | **Poland** |  | **Portugal** |  | **UK** |  | **Sweden** |  |
| --- | --- | --- | --- | --- | --- | --- | --- | --- | --- | --- | --- |
|  |  | **VPT** | **EPT** | **VPT** | **EPT** | **VPT** | **EPT** | **VPT** | **EPT** | **VPT** | **EPT** |
| **Hospital outpatient services** | **Paediatrician** | 3.81 (1.51) | 7.88 (2.29) | 260.31 (27.42) | 294.78 (67.54) | 37.28 (4.05) | 27.97 (4.42) | 52.57 (9.30) | 72.58 (25.33) | 100.52 (31.48) | 118.69 (34.76) |
|  | **A&E** | 14.22 (3.86) | 19.42 (6.37) | 7.23 (1.41) | 3.56 (1.28) | 48.43 (4.37) | 43.90 (5.79) | 27.80 (4.37) | 44.31 (16.44) | 52.79 (11.01) | 115.50 (42.27) |
|  | **Neurologist** | 1.69 (1.30) | 1.34 (1.00) | 24.77 (6.58) | 40.94 (11.65) | 3.73 (1.00) | 4.24 (1.45) | 0.17 (0.05) | 0.35 (0.11) | 19.44 (6.57) | 62.32 (25.02) |
|  | **Ear, Nose and Throat (ENT)** | 5.56 (2.01) | 13.84 (3.41) | 18.47 (3.51) | 19.10 (8.17) | 19.51 (2.28) | 17.46 (2.82) | 4.89 (0.79) | 5.96 (1.42) | 28.71 (7.52) | 43.18 (18.12) |
|  | **Ophthalmologist** | 6.46 (1.70) | 40.73 (20.16) | 29.51 (5.53) | 29.60 (7.59) | 19.07 (1.77) | 21.36 (6.09) | 4.15 (0.51) | 8.03 (1.22) | 46.72 (15.20) | 108.46 (36.81) |
|  | **Speech therapist** | 42.02 (17.60) | 70.65 (25.50) | 547.30 (111.51) | 680.57 (201.42) | 51.18 (9.62) | 54.89 (13.42) | 17.61 (4.85) | 57.82 (26.73) | 77.90 (22.87) | 358.82 (214.76) |
|  | **Psychologist** | 0.00 (0.00) | 4.60 (3.14) | 176.53 (57.62) | 360.50 (167.98) | 13.36 (3.94) | 23.12 (8.73) | 0.65 (0.28) | 7.23 (4.86) | 9.15 (4.28) | 106.99 (44.51) |
|  | **Psychiatrist** | 0.00 (0.00) | 2.42 (1.91) | 4.42 (2.64) | 0.00 (0.00) | 1.44 (0.49) | 1.61 (0.78) | 1.85 (0.77) | 36.81 (31.57) | 9.32 (4.35) | 108.69 (67.42) |
|  | **Physiotherapist** | 34.87 (16.04) | 43.65 (20.44) | 383.96 (125.62) | 566.44 (175.31) | 121.37 (42.24) | 274.30 (98.43) | 39.04 (13.66) | 82.71 (44.18) | 7.57 (2.68) | 138.34 (66.31) |
|  | **Respiratory or asthma specialist** | 1.52 (0.89) | 5.22 (2.67) | 129.44 (23.12) | 104.05 (36.48) | 8.32 (1.60) | 50.51 (40.81) | 2.29 (0.53) | 9.34 (7.38) | 6.39 (1.91) | 13.52 (4.08) |
| **Total Hospital outpatient services** | **Mean (SE)** | 110.15 (33.75) | 209.74 (49.96) | 1581.94 (267.35) | 2099.54 (511.81) | 323.69 (49.40) | 519.36 (117.26) | 151.00 (23.54) | 325.14 (118.87) | 358.51 (57.28) | 1174.49 (358.63) |
|  | **Median (IQR)** | 0.00  (126.8) | 0.00 (0.00) | 0.00 (1295.8) | 260.1 (1511.1) | 0.00 (255.9) | 89.8 (249.2) | 0.00 (0.00) | 0.00 (0.00) | 0.00 (446.4) | 0.00 (421.2) |
| **Hospital inpatient services** | **In hospital days** | 41.08 (19.45) | 89.70 (40.25) | 95.28 (25.60) | 50.64 (20.78) | 54.87 (13.17) | 90.11 (45.39) | 23.81 (9.18) | 58.75 (22.74) | 35.13 (15.93) | 581.23 (228.48) |
|  | **HIS (Median, (IQR)** | 0.00  (0.00) | 0.00  (0.00) | 0.00 (0.00) | 0.00 (0.00) | 0.00 (0.00) | 0.00 (0.00) | 0.00 (0.00) | 0.00 (0.00) | 0.00 (0.00) | 0.00 (0.00) |
| **Community health and social services** | **GP** | 12.75  (2.23) | 23.57 (3.75) | 152.97 (22.59) | 140.58 (38.27) | 13.74 (1.53) | 10.54 (2.38) | 26.04 (2.81) | 23.63 (3.59) | 34.13 (5.46) | 34.92 (9.12) |
|  | **Dietician** | 0.88  (0.56) | 1.01 (0.84) | 2.09 (1.11) | 0.00 (0.00) | 1.13 (0.39) | 1.02 (0.48) | 1.85 (0.62) | 7.75 (2.38) | 8.70 (3.21) | 16.55 (8.04) |
|  | **Health visitor** | 0.76  (0.76) | 2.83 (2.83) | 2.38 (0.97) | 3.54 (3.05) | 0.93 (0.41) | 17.40 (10.15) | 2.55 (0.98) | 1.57 (0.73) | 0.00 (0.00) | 0.00 (0.00) |
|  | **School Nurse** | 4.41  (0.90) | 2.11 (0.78) | 2.47 (0.81) | 1.15 (0.66) | 1.84 (0.62) | 0.96 (0.40) | 5.31 (1.25) | 10.88 (4.48) | 0.00 (0.00) | 0.00 (0.00) |
|  | **Occupational therapist** | 0.99  (0.91) | 6.33 (4.76) | 328.55 (115.82) | 277.11 (98.50) | 26.58 (6.85) | 49.16 (13.56) | 34.47 (14.90) | 80.21 (60.28) | 19.30 (7.93) | 398.05 (324.50) |
|  | **Early childhood intervention** | 0.00  (0.00) | 1.47 (1.47) | 294.61 (139.81) | 156.81 (62.58) | 77.58 (16.73) | 144.56 (40.32) | 904.65 (557.46) | 844.64 (475.15) | 40.49 (17.51) | 104.94 (47.51) |
|  | **Others** | 9.94  (1.26) | 7.55 (1.53) | 55.64 (5.90) | 31.99 (6.11) | 6.34 (0.79) | 7.12 (1.75) | 1.41 (0.34) | 1.79 (0.59) | 92.56 (16.50) | 143.38 (30.83) |
| **Total Community health and social services** | **Mean (SE)** | 29.74  (3.44) | 44.88 (7.46) | 838.72 (230.99) | 611.18 (151.62) | 128.13 (20.99) | 230.76 (56.49) | 976.29 (567.49) | 970.48 (480.51) | 195.17 (31.64) | 697.85 (336.84) |
|  | **Median (IQR)** | 0.00  (58.1) | 0.00 (29.0) | 0.00 (255.5) | 132.6 (369.3) | 0.00 (34.2) | 0.00 (35.9) | 0.00 (0.00) | 0.00 (0.00) | 0.00 (294.2) | 0.00 (150.4) |
| **Equipment costs¹** | **Mean (SE)** | 7.31  (4.23) | 6.14 (3.19) | 196.01 (105.00) | 226.45 (87.27) | 24.99 (6.58) | 6.91 (2.23) | 12.21 (10.02) | 12.85 (10.38) | 16.76 (10.11) | 620.73 (585.48) |
|  | **Median (IQR)** | 0.00  (0.00) | 0.00 (0.00) | 0.00 (0.00) | 0.00 (0.00) | 0.00 (0.00) | 0.00 (0.00) | 0.00 (0.00) | 0.00 (0.00) | 0.00 (0.00) | 0.00 (0.00) |
| **Out of pocket expenses** | **Mean (SE)** | 5.61  (3.45) | 17.39 (14.76) | 644.27 (180.29) | 875.73 (323.45) | 89.35 (23.62) | 75.00 (32.09) | 34.23 (25.06) | 14.81 (8.25) | 80.89 (54.47) | 4440.24 (2751.18) |
|  | **Median (IQR)** | 0.00  (0.00) | 0.00 (0.00) | 0.00 (0.00) | 0.00 (300.0) | 0.00 (0.00) | 0.00 (0.00) | 0.00 (0.00) | 0.00 (0.00) | 0.00 (0.00) | 0.00 (0.00) |
| **Valuation of time off work** | **Mean (SE)** | 356.53 (268.43) | 61.31 (29.13) | 854.05 (308.89) | 804.05 (379.13) | 706.00 (186.02) | 789.79 (252.89) | 285.52 (113.19) | 342.68 (149.96) | 816.91 (569.04) | 1266.33 (503.26) |
|  | **Median (IQR)** | 0.00  (0.00) | 0.00 (0.00) | 0.00 (0.00) | 0.00 (300.0) | 0.00 (0.00) | 0.00 (0.00) | 0.00 (0.00) | 0.00 (0.00) | 0.00 (0.00) | 0.00 (0.00) |
| **Total societal costs^2^** | **Mean (SE)** | 550.42 (300.19) | 429.18 (90.38) | 4210.26 (733.99) | 4667.59 (1055.50) | 1327.03 (235.03) | 1711.93 (397.31) | 1483.06 (670.33) | 1724.70 (560.54) | 1503.37 (626.89) | 8780.88 (3317.96) |
|  | **Median (IQR)** | 0.00  (306.4) | 0.00 (55.2) | 0.00 (3305.1) | 589.5 (3183.4) | 0.00 (500.9) | 131.3 (472.4) | 0.00 (0.00) | 0.00 (0.00) | 14.8 (2711.8) | 73.9 (793.7) |

¹ These costs include any special equipment related to children such as wheelchair, brace, hearing aids or shoes.

² Sum of all the cost categories.

A&E: Accident and Emergency; GP: General Practitioner

**Appendix 4: Resource use values and unit costs for resource inputs for children with complete data by country and study period over 5 years, mean (SE) (€, 2016 prices)**

| **Mean (SE)** | **Belgium** | **Denmark** | **Estonia** | **France** | **Germany** | **Italy** | **Netherlands** | **Poland** | **Portugal** | **UK** | **Sweden** |
| --- | --- | --- | --- | --- | --- | --- | --- | --- | --- | --- | --- |
| **Paediatrician (Visit,** Mean (SE) | 0.35 (0.06) | 0.18  (0.04) | 0.56 (0.12) | 0.39 (0.03) | 1.07  (0.10) | 1.90 (0.16) | 0.13  (0.03) | 3.21 (0.37) | 0.61 (0.06) | 0.19  (0.03) | 0.49 (0.12) |
| **Unit cost (€ per visit)** | 37.48 | 52.06 | 28.13 | 22.68 | 18.94 | 22.56 | 28.6 | 67.1 | 41.53 | 234.07 | 173.79 |
| **GP (Visit** Mean (SE) | 0.73 (0.07) | 0.49  (0.06) | 2.24 (0.27) | 1.10 (0.08) | 0.18  (0.04) | 0.14 (0.02) | 0.41  (0.05) | 1.77 (0.24) | 0.52 (0.05) | 0.48  (0.04) | 0.40 (0.06) |
| **Unit cost (€ per visit)** | 25 | 52.06 | 7 | 23.12 | 37.64 | 20.37 | 29.04 | 66.32 | 17.93 | 42.34 | 73.42 |
| **A&E (Visit** Mean (SE) | 0.07 (0.01) | 0.05  (0.01) | 0.23 (0.05) | 0.16 (0.02) | 0.17  (0.03) | 0.27 (0.03) | 0.07  (0.02) | 0.24 (0.04) | 0.73 (0.06) | 0.11  (0.02) | 0.19 (0.04) |
| **Unit cost (€ per visit)** | 20.21 | 134 | 33.47 | 144.43 | 155.12 | 52.75 | 157.65 | 18.94 | 48.23 | 245.83 | 295.96 |
| **Neurologist (Visit** Mean (SE) | 0.05 (0.01) | 0.02  (0.01) | 0.79 (0.56) | 0.10 (0.06) | 0.06  (0.02) | 0.14 (0.02) | 0.02  (0.01) | 0.35 (0.07) | 0.05 (0.01) | 0.02  (0.00) | 0.15 (0.04) |
| **Unit cost (€ per visit)** | 68.27 | 62.5 | 28.13 | 36.08 | 35.77 | 23.08 | 61.61 | 69.42 | 41.53 | 11.36 | 189.27 |
| **ENT (Visit** Mean (SE) | 0.15 (0.02) | 0.27  (0.04) | 0.40 (0.08) | 0.19 (0.03) | 0.36  (0.04) | 0.23 (0.02) | 0.10  (0.02) | 0.49 (0.10) | 0.34 (0.03) | 0.09  (0.01) | 0.21 (0.05) |
| **Unit cost (€ per visit)** | 27.64 | 62.5 | 18.49 | 29.9 | 18.94 | 23.08 | 61.61 | 29.41 | 41.53 | 46.6 | 126.45 |
| **Ophthalmologist (Visit** Mean (SE) | 0.21 (0.06) | 0.12  (0.03) | 0.46 (0.07) | 0.54  (0.06) | 0.36  (0.03) | 0.38 (0.03) | 0.31  (0.12) | 0.59 (0.09) | 0.36 (0.04) | 0.16  (0.02) | 0.43 (0.11) |
| **Unit cost (€ per visit)** | 25 | 62.5 | 28.13 | 27.96 | 59.27 | 23.08 | 42.26 | 37.64 | 41.53 | 25.73 | 125.26 |
| **Speech therapist (Visit** Mean (SE) | 1.90 (0.44) | 0.39  (0.14) | 4.52 (1.08) | 2.38 (0.27) | 2.39  (0.34) | 4.01 (0.47) | 1.08  (0.30) | 7.02 (1.25) | 3.96 (0.64) | 0.47  (0.14) | 0.42 (0.12) |
| **Unit cost (€ per visit)** | 44.47 | 33.02 | 34.34 | 51.65 | 32.55 | 9.41 | 34.45 | 64.79 | 10.45 | 49.4 | 150.12 |
| **Psychologist (Visit** Mean (SE) | 0.15 (0.05) | 0.09  (0.04) | 0.49 (0.22) | 0.63 (0.12) | 0.17  (0.13) | 0.26 (0.07) | 0.01  (0.01) | 3.25 (0.96) | 0.76 (0.18) | 0.03  (0.02) | 0.22 (0.08) |
| **Unit cost (€ per visit)** | 50 | 62.50 | 162.8 | 23.1 | 55.12 | 23.08 | 79.35 | 59.24 | 15.27 | 61.16 | 153.91 |
| **Psychiatrist (Visit** Mean (SE) | 0.01 (0.00) | 0.00  (0.00) | 0.02 (0.01) | 0.07 (0.03) | 0.03  (0.02) | 0.10 (0.03) | 0.01  (0.01) | 0.01 (0.01) | 0.03 (0.01) | 0.06 (0.05) | 0.12 (0.06) |
| **Unit cost (€ per visit)** | 47.08 | 126.26 | 28.13 | 170.1 | 18.94 | 21.64 | 83.52 | 175.86 | 32.82 | 162.32 | 287.49 |
| **Physiotherapist (Visit** Mean (SE) | 2.61 (0.47) | 1.22  (0.54) | 3.42 (1.07) | 2.24(0.3) | 1.01  (0.27) | 5.05 (0.61) | 0.75  (0.25) | 7.86 (1.76) | 2.00 (0.50) | 0.38  (0.12) | 0.62 (0.30) |
| **Unit cost (€ per visit)** | 22.26 | 33.98 | 16.6 | 30.16 | 21.06 | 11.36 | 36.29 | 46.28 | 64.31 | 110.56 | 36.83 |
| **Respiratory specialist (Visit** Mean (SE) | 0.04 (0.01) | 0.02  (0.01) | 0.19 (0.04) | 0.05 (0.01) | 0.02  (0.01) | 0.09 (0.01) | 0.04  (0.01) | 0.44 (0.08) | 0.38 (0.25) | 0.09  (0.05) | 0.10 (0.02) |
| **Unit cost (€ per visit)** | 39.3 | 62.5 | 28.13 | 63.84 | 26.99 | 23.08 | 55.39 | 176.43 | 41.53 | 37.96 | 73.92 |
| **Dietician (Visit** Mean (SE) | 0.02 (0.01) | 0.04  (0.02) | 0.02 (0.01) | 0.00 (0.00) | 0.01  (0.01) | 0.03 (0.01) | 0.02  (0.01) | 0.02 (0.01) | 0.02 (0.01) | 0.06  (0.01) | 0.19 (0.06) |
| **Unit cost (€ per visit)** | 19.66 | 31.5 | 28.13 | 61.4 | 21.06 | 23.08 | 28 | 46.28 | 41.53 | 49.4 | 50.27 |
| **Health visitor (Visit** Mean (SE) | 0.00 (0.00) | 0.05  (0.02) | 0.67 (0.21) | 0.00 (0.00) | 0.02  (0.01) | 0.30 (0.23) | 0.02  (0.01) | 0.07 (0.03) | 0.11 (0.06) | 0.03  (0.01) | 0.00 (0.00) |
| **Unit cost (€ per visit)** | 29.6 | 29.6 | 18.5 | 18.8 | 20.5 | 19.4 | 65 | 29.6 | 44.4 | 50.6 | 29.6 |
| **School nurse (Visit** Mean (SE) | 0.08 (0.03) | 0.00  (0.00) | 0.20 (0.08) | 0.00 (0.00) | 0.00  (0.00) | 0.00 (0.00) | 0.06  (0.01) | 0.07 (0.02) | 0.05 (0.01) | 0.11  (0.03) | 0.00 (0.00) |
| **Unit cost (€ per visit)** | 4 | 30.77 | 2.83 | 18.78 | 34.55 | 10.96 | 41.64 | 22.37 | 21.44 | 50.58 | 119.66 |
| **Occupational therapist (Visit** Mean (SE) | 0.58 (0.21) | 0.32  (0.14) | 0.75 (0.33) | 8.87  (6.14) | 1.92  (0.31) | 0.24 (0.12) | 0.09  (0.06) | 3.58 (1.03) | 2.38 (0.44) | 0.24  (0.10) | 0.12 (0.04) |
| **Unit cost (€ per visit)** | 49.4 | 33.1 | 12 | 2.1 | 43.6 | 4.9 | 23 | 59.6 | 10.9 | 162.3 | 255 |
| **Early childhood intervention (Visit** Mean (SE) | 0.18 (0.11) | 0.28  (0.24) | 0.60 (0.56) | 0.00 (0.00) | 0.89  (0.22) | 0.61 (0.18) | 0.01  (0.01) | 2.73 (1.06) | 1.83 (0.33) | 0.08  (0.04) | 0.33 (0.10) |
| **Unit cost (€ per visit)** | 62.8 | 67.7 | 16.74 | 67.7 | 20.5 | 22.34 | 67.7 | 67.7 | 39.66 | 152.2 | 159.36 |
| **In hospital (Day, mean(SE)** | 0.09 (0.02) | 0.04  (0.03) | 0.29 (0.10) | 0.12 (0.02) | 0.29  (0.06) | 0.24 (0.04) | 0.09  (0.03) | 0.58 (0.13) | 0.14 (0.04) | 0.08  (0.02) | 0.24 (0.09) |
| **Unit cost (€ per night)** | 611.33 | 915.11 | 86.42 | 620.3 | 769.46 | 2326.83 | 476.12 | 107.73 | 350.42 | 352.51 | 722.14 |
| **Lost working days due to childcare (Day, mean(SE)** | 3.36 (0.95) | 4.1  (1.77) | 9.07 (1.84) | 1.55 (0.34) | 1.38  (0.5) | 5.06 (0.91) | 1.4 (0.97) | 9.92 (2.84) | 9.02 (1.84) | 2.1 (0.63) | 6.7  (2.97) |
| **Unit cost (€ per day)** | 174.99 | 177.87 | 80.8 | 142.62 | 161.42 | 121.89 | 180.03 | 84.22 | 81.37 | 144.11 | 142.44 |
| **Other health services use^1^** | 0.04(0.01) | 0.03(0.01) | 0.29(0.04) | 0.01(0) | 0.1(0.01) | 0(0) | 0.18(0.02) | 0.44(0.04) | 0.13(0.01) | 0.01(0) | 0.28(0.03) |
| ^1^ This includes various types of services in a free-text format. | | | | | | | | | | | |

**Appendix 5 Multivariate analysis to examine the association between total societal costs at 5 years of age and infant’s health status, maternal socioeconomic status, and multiple status (Generalised Linear Model); gestational age presented in four categories (€, 2016 prices)**

|  |  | **Belgium** | **Denmark** | **Estonia** | **France** | **Germany** | **Italy** | **Netherlands** | **Poland** | **Portugal** | **UK** | **Sweden** |
| --- | --- | --- | --- | --- | --- | --- | --- | --- | --- | --- | --- | --- |
| **Gestational age (ref : 30-31 week)** | **<26** | 1056.61 | 6038.34 | -306.14 | 2065.64*** | 1567.82 | -1363.05** | 699.94 | 5476.25* | 2458.01 | 22176.83*** | 3201.59 |
|  |  | (1486.73) | (3629.12) | (856.85) | (624.43) | (1127.17) | (431.66) | (456.35) | (2611.07) | (1306.19) | (5412.38) | (2331.81) |
|  | **26-27** | -1105.62* | 989.96 | -387.84 | 774.25*** | 76.58 | 1534.79** | 504.64* | 6161.18** | 1839.38*** | -761.61 | -1652.89 |
|  |  | (462.76) | (729.31) | (430.48) | (225.26) | (672.03) | (539.55) | (253.38) | (2304.54) | (531.45) | (413.04) | (1262.08) |
|  | **28-29** | 1096.38 | -378.56 | 427.98 | 218.32 | 1863.85* | -611.63* | 1761.38*** | 3145.58 | 226.22 | 1994.83** | -1491.21 |
|  |  | (753.75) | (466.59) | (560.87) | (141.15) | (845.00) | (287.71) | (525.62) | (1759.89) | (259.32) | (631.33) | (1012.38) |
| **SGA (ref>10^th^ centile)** | **<=10^th^ centile** | 3239.92** | 223.77 | 0.30 | 351.77* | 1022.18 | -79.59 | 744.22 | -1483.55 | 1687.86*** | 1043.24 | 1608.03 |
|  |  | (1205.92) | (692.14) | (402.18) | (169.59) | (606.56) | (288.45) | (424.14) | (1571.42) | (438.92) | (1207.51) | (1511.54) |
| **BPD (ref: none)** | **Yes** | 3601.02* | -913.10 | -328.06 | 789.31* | 4049.17* | 2898.96** | 498.60 | 5971.35* | 805.62 | -936.19 | 141.60 |
|  |  | (1735.09) | (812.07) | (560.73) | (307.07) | (2062.18) | (945.36) | (444.32) | (2934.76) | (597.13) | (1239.97) | (1448.68) |
| **Severe, congenital anomalies (ref: none)** | **Yes** | 21192.49 | -1956.70** | 2844.53 | 419.43 | 2512.76 | 5644.28*** | 192.13 | 5864.10** | -583.66 | 1220.36 | 5624.93 |
|  |  | (14709.72) | (597.23) | (2349.03) | (307.72) | (1731.53) | (1502.11) | (504.23) | (1892.34) | (544.46) | (2097.52) | (3272.53) |
| **Any**  **morbidity (ref: none)** | **Neonatal morbidity at discharge** | 1082.57 | 3103.09 | 686.52 | 518.08 | 2927.31 | 5050.08*** | -161.57 | 5088.81** | 3098.36*** | 8460.65*** | 11311.56* |
|  |  | (1270.19) | (2947.30) | (935.12) | (377.00) | (1850.05) | (1168.39) | (427.39) | (1881.82) | (936.18) | (2455.77) | (5222.15) |
| **Sex (ref: Female)** | **Male** | 222.96 | 3389.32* | 1295.42*** | 561.57*** | 873.86 | 523.08 | 1646.35*** | 4153.17** | 1055.48*** | 6009.37*** | 588.80 |
|  |  | (498.19) | (1442.00) | (345.52) | (142.48) | (598.20) | (276.33) | (385.18) | (1397.29) | (309.83) | (1294.95) | (1056.87) |
| **One or both parents unemployed (ref: employed)** | **Unemployed** | 6898.02 | 10642.76 | 2551.04 | -464.25** | 235.42 | -1235.48*** | -705.44 | -3653.07 | 3950.40*** | -2480.61 | 7153.78 |
|  |  | (8207.62) | (10912.75) | (1507.44) | (159.24) | (3453.40) | (343.69) | (396.04) | (2036.49) | (849.36) | (1537.39) | (7728.27) |
| **Maternal education (ref : higher education)** | **High school or less** | -804.31 | 806.35 | -52.77 | -237.03 | 1162.53 | 1726.99*** | 439.81 | -2415.13 | 452.98 | 553.13 | 4917.32** |
|  |  | (525.29) | (757.13) | (354.96) | (158.35) | (611.25) | (291.26) | (271.14) | (1571.43) | (304.92) | (984.84) | (1582.89) |
| **Country of birth for mothers (ref : native)** | **European born** | -2543.05*** | -539.68 | 4491.67 | 1339.12 | -1151.39 | -131.25 | -1561.65*** |  | -1718.47*** |  | -1278.70 |
|  |  | (432.43) | (1603.51) | (7144.30) | (956.37) | (659.05) | (398.04) | (297.65) |  | (355.90) |  | (1943.90) |
|  | **non-European born** | -2301.96*** | -1452.50 |  | -97.12 | 3761.87 | -629.38 | -1108.83*** |  | -263.49 | -4646.76*** | -3870.45** |
|  |  | (545.20) | (1200.45) |  | (148.29) | (2581.83) | (446.36) | (297.06) |  | (539.98) | (1099.19) | (1195.78) |
| **Maternal age (ref 25-34)** | **<25** | -1777.30*** | -416.59 | -876.04* | 343.57 | 669.84 | -1188.33*** | 3383.63* | 2808.79 | -187.79 | 11624.57** | -4807.87*** |
|  |  | (471.22) | (780.74) | (413.35) | (245.37) | (1305.48) | (249.29) | (1676.51) | (1809.94) | (508.12) | (3589.37) | (1240.75) |
|  | **>34** | 2302.73 | 2379.22* | -273.45 | 109.44 | 998.57 | 1870.31*** | 30.82 | 7889.10** | -455.37 | 3588.71** | -778.79 |
|  |  | (1269.63) | (1192.36) | (414.44) | (179.17) | (852.97) | (357.03) | (263.97) | (2553.51) | (322.36) | (1242.04) | (1295.35) |
| **First child (ref: not the first child)** |  | -62.29 | 218.25 | 33.09 | -57.93 | 873.30 | -352.95 | -998.78 | 1373.39 | 567.68 | -8108.42*** | 887.90 |
|  |  | (503.13) | (648.37) | (334.28) | (145.73) | (628.94) | (317.01) | (557.32) | (1534.97) | (289.71) | (1989.84) | (1131.55) |
| **Multiple (ref: singleton)** | **Twins** | -564.79 | -1162.56 | -558.24 | -199.80 | 195.55 | -1640.70*** | -466.28 | 3321.77 | -246.70 | -3814.10*** | -1090.62 |
|  |  | (486.64) | (851.88) | (411.75) | (142.44) | (735.60) | (277.08) | (278.33) | (1722.69) | (329.81) | (878.08) | (1002.55) |
|  | **Triplets or quadruplets** |  | -1852.68* | -1536.76*** | 778.48 | -197.91 | -164.82 | -1138.98* | -1064.97 | -136.85 | -1718.17 | 764.54 |
|  |  |  | (896.89) | (252.65) | (1022.19) | (1062.50) | (717.26) | (530.37) | (5768.73) | (1036.37) | (1691.00) | (3564.71) |

SGA: small for gestational age

BPD: bronchopulmonary dysplasia

Morbidity: Intraventricular haemorrhage grades III-IV (IVH), periventricular leukomalacia (PVL), retinopathy of prematurity stages III-V (ROP) or necrotising enterocolitis needing surgery (NEC)

| Standard errors in parentheses | | |
| --- | --- | --- |
| * p<0.05 | **p<0.01 | ***p<0.001 |

**Appendix 6: Multilevel analysis excluding employment status (€, 2016 prices) at 5 years of age (Generalised Linear Model)**

| ***Perinatal characteristics*** | | **Total costs per child** | **Total societal costs per child** |
| --- | --- | --- | --- |
| **Gestational age (ref 30-31 weeks)** | **<26** | 2475.84*** | 3835.21*** |
|  |  | (713.97) | (1055.66) |
|  | **26-27** | 663.93*** | 1231.81*** |
|  |  | (199.28) | (357.27) |
|  | **28-29** | 358.33** | 651.23** |
|  |  | (121.00) | (227.20) |
| **SGA (ref>10^th^ centile)** | **<=10^th^ centile** | 272.32* | 352.88 |
|  |  | (122.35) | (220.77) |
| **BPD (ref: none) Yes** | |  |  |
|  |  |  |  |
| **Severe congenital anomalies (ref: none)** | **Yes** |  |  |
|  |  |  |  |
| **Any morbidity (ref: none)** | **Yes** |  |  |
|  |  |  |  |
| **Sex (ref: Female)** | **Male** | 378.22** | 837.26*** |
|  |  | (121.50) | (233.68) |
| ***Sociodemographic characteristics*** | | | |
| **Household employment status(ref: employed)** | **At least one parent unemployed** |  |  |
| **Maternal education (ref : higher education)** | **High school or less** | 149.35 | 310.62 |
|  |  | (102.76) | (203.99) |
| **Country of birth for mothers (ref : native)** | **European born** | -339.87 | -713.47* |
|  |  | (186.65) | (342.57) |
|  | **non-European born** | -575.47*** | -712.90** |
|  |  | (151.82) | (251.27) |
| **Mother age at childbirth (ref 25-34 years)** | **<25** | 106.20 | 419.72 |
|  |  | (151.70) | (334.23) |
|  | **>34** | 167.59 | 364.38 |
|  |  | (116.80) | (231.65) |
| **Parity (ref: multiparous)** | | -155.96 | -25.76 |
|  |  | (106.60) | (194.27) |
| **Multiple (ref: singleton)** | **Twins** | -109.87 | -313.47 |
|  |  | (98.42) | (202.95) |
|  | **Triplets or quadruplets** | -79.92 | -885.85* |
|  |  | (228.99) | (344.39) |

**Appendix 7:** **Multilevel analysis using inverse probability weighting (IPW) to examine the association between total costs (€, 2016 prices) at 5 years of age and children’s health status, mothers’ socioeconomic status, and multiple status (Generalised Linear Model)**

|  |  | **Total societal costs per child** | **Total societal costs using inverse probability weighting** |
| --- | --- | --- | --- |
| ***Perinatal characteristics*** | |  |  |
| **Gestational age (ref 30-31 weeks)** | **<26** | 2755.98*** | 2738.11 |
|  |  | (707.71) | (1663.00) |
|  | **26-27** | 752.34** | 762.42* |
|  |  | (282.32) | (355.69) |
|  | **28-29** | 657.09** | 671.17 |
|  |  | (233.56) | (355.82) |
| **SGA (ref>10^th^ centile)** | **<10^th^ centile** | 307.90 | 323.62 |
|  |  | (228.03) | (360.73) |
| **BPD (ref: none) Yes** | | 1128.05** | 1142.29 |
|  |  | (408.98) | (662.26) |
| **Congenital anomalies (ref: none)** | **Yes** | 3112.72*** | 3042.43* |
|  |  | (773.21) | (1374.86) |
| **Any morbidity (ref: none)** | **Severe neonatal morbidity at discharge** | 3233.58*** | 3199.79*** |
|  |  | (697.97) | (856.35) |
| **Sex (ref: Female)** | **Male** | 1226.62*** | 1233.52*** |
|  |  | (243.35) | (357.67) |
| ***Sociodemographic characteristics*** | | | |
| **Household employment status(ref: employed)** | **At least one parent unemployed** | 426.28 | 441.06 |
|  |  | (378.60) | (952.28) |
| **Mom education (ref : higher education)** | **High school or less** | 625.17** | 639.24 |
|  |  | (218.60) | (389.83) |
| **Country of birth for mothers (ref : native)** | **European born** | -477.84 | -489.12 |
|  |  | (428.14) | (438.16) |
|  | **non-European born** | -577.29* | -566.25 |
|  |  | (282.16) | (384.40) |
| **Mother age at childbirth (ref 25-34 years)** | **<25** | 493.15 | 493.22 |
|  |  | (368.39) | (680.22) |
|  | **>34** | 671.45** | 666.22 |
|  |  | (259.00) | (366.38) |
| **Parity (ref: multiparous)** | | -52.68 | -45.65 |
|  |  | (211.56) | (385.23) |
| **Multiple (ref: singleton)** | **Twin** | -383.66 | -390.61 |
|  |  | (213.93) | (204.09) |
|  | **Triplet&+** | -809.38 | -807.60 |
|  |  | (439.62) | (522.14) |

**Appendix 8: Percentage of live births by gestational age in weeks**

|  |  | % of live births |
| --- | --- | --- |
|  |  | <32 weeks |
| Belgium |  | 1.1 |
| Denmark |  | 0.9 |
| Estonia |  | 1.1 |
| France |  | 1.1 |
| Germany |  | 1.3 |
| Italy |  | 1.0 |
| Netherlands | | 1.1 |
| Poland |  | 1.1 |
| Portugal |  | 1.0 |
| U.K |  | 1.2 |
| Sweden |  | 0.9 |

Source: EURO-peristat report [39]
